# CAR‐NK cells: the next wave of cellular therapy for cancer

**DOI:** 10.1002/cti2.1274

**Published:** 2021-04-28

**Authors:** May Daher, Luciana Melo Garcia, Ye Li, Katayoun Rezvani

**Affiliations:** ^1^ Department of Stem Cell Transplantation and Cellular Therapy The University of Texas MD Anderson Cancer Center Houston TX USA

**Keywords:** allogeneic, cancer immunotherapy, CAR, cellular therapy, NK cells, off‐the‐shelf product

## Abstract

T cells engineered to express chimeric antigen receptors (CARs) have revolutionised the field of cellular therapy for cancer. Despite its success, this strategy has some recognised limitations and toxicities. Hence, there is growing interest in developing novel cellular therapies based on non‐αβ T‐cell immune effector cells, including NK cells that offer clear advantages in cancer immunotherapy. As a result, NK cells are being explored as an alternative platform for CAR engineering and are becoming recognised as important players in the next generation of cellular therapies targeting cancer. In this review, we highlight preclinical and clinical studies of CAR‐NK cells derived from different sources and discuss strategies under investigation to enhance the antitumor activity of these engineered innate immune cells.

## Graphical abstract

NK cells can be derived from various sources (PB, UCB, HSC, iPSC, NK cell lines) and can be engineered to express a chimeric antigen receptor (CAR) to target various surface antigens on cancer cells. These CAR‐NK cells can be used as off‐the‐shelf adoptive cellular therapy to treat patients with various malignancies.

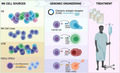

## Introduction

This past decade has seen impressive advances in the cellular therapy field. Autologous T cells genetically engineered to express a chimeric antigen receptor (CAR) have induced durable clinical responses in patients with B‐cell acute lymphoblastic leukaemia (B‐ALL) or B‐cell lymphomas as two striking examples.^1^ Despite this clinical success, manufacturing complexities and severe toxicities are persistent limitations of CAR T‐cell therapy.^2^ These challenges highlight the need to investigate alternative immune effector cells as potential vehicles for CAR engineering with an improved safety profile and the potential to be used as allogeneic off‐the‐shelf products.

CARs are engineered receptor molecules that direct immune effector cell functions through specific antigen recognition by its immunoglobulin‐like extracellular domain, the single‐chain variable fragment (scFv).^3^ Once equipped with a CAR, T cells are redirected to recognise and kill tumor cells expressing the target antigen.^4^ Thus far, four cellular therapies, all CD19‐targeted T‐cell products, have been approved by the Food and Drug Administration (FDA): tisagenlecleucel (KYMRIAH, Novartis), axicabtagene ciloleucel (Yescarta, Kite Pharma) and brexucabtagene autoleucel (KTE‐X19, Tercartus). Tisagenlecleucel was shown to be effective in children and young adults with B‐ALL and adult patients with aggressive B‐cell lymphomas.^5^, ^6^ Axicabtagene ciloleucel is approved for the treatment of patients with relapsed or refractory aggressive B‐cell lymphomas.^7^ Recently, brexucabtagene autoleucel was shown to have impressive activity in patients with relapsed or refractory mantle cell lymphoma.^8^ Additionally, a fourth CD19‐targeted product, lisocabtagene maraleucel (Juno Therapeutics), was just granted FDA approval for the treatment of relapsed or refractory large B‐cell lymphomas.^9^ CAR T‐cell therapies targeting various other antigens are also under investigation for haematologic malignancies, including T‐cell leukaemias and lymphomas, myeloid malignancies and multiple myeloma.

Large‐scale use of CAR T‐cell therapy (Table 1) faces a number of challenges. First of all, treatment with these modified cells can cause serious adverse events, such as cytokine release syndrome (CRS) and immune effector cell‐associated neurotoxicity syndrome (ICANS), which may increase the length of hospitalisation and raise the cost of therapy.^10^ Second, manufacturing of autologous CAR T cells can be a cumbersome, expensive and lengthy procedure,^11^ as it involves the collection of T cells from heavily pretreated patients, their transduction and expansion, and finally infusion of the CAR T products.^11^ Given these shortcomings, there is growing interest in other subsets of immune effectors, such as natural killer (NK) cells. NK cells represent an essential component of the innate immune system and play an important role in the first line of defence against pathogens and cancer.^12^ As their name indicates, NK cells are specialised killers with the natural ability to eliminate abnormal cells without the need for prior sensitisation, as they rely on the balance between activating and inhibitory signals from germline‐encoded receptors.^12^ Unlike allogeneic T cells, allogeneic NK cells do not cause graft‐versus‐host disease and thus pave the way for a potentially off‐the‐shelf product for cell therapy.

**Table 1:** Clinical trials for CD19‐directed CAR T cell therapy

Clinical trial	ELIANA trial (NCT02435849)	JULIET trial (NCT02445248)	ZUMA‐1 trial (NCT02348216)	TRANSCEND NHL 001 trial (NCT 02631044)	ZUMA‐2 trial (NCT02601313)
Drug	Tisagenlecleucel (Kymriah, Novartis)	Tisagenlecleucel (Kymriah, Novartis)	Axicabtagene ciloleucel (Yescarta, Kite)	Lisocabtagene maraleucel (Juno Therapeutics)	Axicabtagene ciloleucel KTE‐X19 (Yescarta, Kite)
Target	CD19	CD19	CD19	CD19	CD19
Stimulatory and co‐stimulatory domains	CD3 ζ and 1‐4BB	CD3 ζ and 1‐4BB	CD3 ζ and CD28	CD3 ζ and 1‐4BB	CD3 ζ and CD28
Study design	Single‐arm, multicentered, unblinded phase 2	Single‐arm, multicenter, open labeled, phase 2	Single‐arm, multicenter, unblinded, phase 1–2	Single‐arm, multicenter, multicohort, unblinded, phase 1–2	Multicenter, phase 2 trial
Population	Patients within 3–23 years old with R/R B‐ALL	Patients 18 years or older with R/R DLBCL who received at least 2 prior lines of therapy (*de novo* DLBCL or from transformed FL, and high‐grade B‐cell lymphoma with *MYC* rearrangement plus rearrangement of *Bcl2*, *Bcl6* or both)	Patients 18 years or older with refractory large B cell lymphoma (DLBCL, transformed follicular lymphoma, primary mediastinal B cell lymphoma)	Patients 18 years or older with R/R large B‐cell lymphoma (transformed or *de novo* DLBCL, primary mediastinal B cell lymphoma and high‐grade B‐cell lymphoma with *MYC* rearrangement plus rearrangement of *Bcl2*, *Bcl6* or both), FL grade 3B or relapsed MCL	Patients 18 years or older with R/R mantle cell lymphoma
Previous lines of treatment, range (median)	1–8 (3)	1–8 (not reported)	2–4 (3)	2–4 (3)	1–5 (3)
Previous autologous stem cell transplantation, *n* (%)	46 (61)	18 (33)	Not reported	90 (33)	29 (43)
Lymphodepletion chemotherapy	Fludarabine 30 mg/m^2^ IV (daily doses for 4 doses) and cyclophosphamide 500 mg/m^2^ IV (daily doses for 2 doses)	Fludarabine 25 mg/m^2^ IV (daily doses for 3 days) and cyclophosphamide 250 mg/m^2^ IV (daily doses for 3 doses) or bendamustine 90 mg/m^2^ IV (daily doses for 2 days)	Fludarabine 30 mg/m^2^ IV (daily doses for 3 days) and cyclophosphamide 500 mg/m^2^ IV (daily doses for 3 doses	Fludarabine 30 mg/m^2^ IV (daily doses for 3 doses) and cyclophosphamide 300 mg/m^2^ IV (daily doses for 3 doses)	Fludarabine 30 mg/m^2^ IV (daily doses for 3 days) and cyclophosphamide 500 mg/m^2^ IV (daily doses for 3 doses
T cell dose	Single dose of 0.2–5 × 10^6^ cells/kg (patients < or = 50 kg) or 0.1–2.5 × 10^6^ cells/kg (patients > 50 kg)	Single dose of 0.1–6 × 10^8^ cells (median 3 × 10^8^ cells)	Single dose of 2 × 10^6^ cells/kg (minimum 1 × 10^6^ cells/kg)	Sequential infusions DL 1: 25 × 10^6^ CD8^+^cells and 25 × 10^6^ CD4^+^ cells (on day 1 only) DL 1D: 25 × 10^6^ CD8^+^ cells and 25 × 10^6^ CD4^+^ cells (on day 1 and on day 15) DL2: 50 × 10^6^ CD8^+^ cells and 50 × 10^6^ CD4^+^ cells DL 3: 100 × 10^6^ CD8^+^ cells and 100 × 10^6^ CD4^+^ cells (Median 91 × 10^6^ T cells; range 44–156 × 10^6^ T cells)	Single dose of 2 × 10^6^ cells/kg (minimum 1 × 10^6^ cells/kg)
Primary endpoints	Overall remission rate (CR/CRi) by month 3	Best overall response rate (CR + PR)	Objective response (CR + PR)	Best overall response rate (CR + PR), incidence of adverse events and probability of dose‐limiting toxicities	Objective response (CR+ PR)
Response rates	ORR 81% (CR 60% and CRi 21%)	Best ORR 52% (CR 40% and PR 12%)	Objective response 83% (CR 58% and PR 25%)	Objective response DL 1: 67.5% (CR 60%) DL 2: 74% (CR 52.1%) DL 3: 73.2% (CR 51.2%)	Objective response 93% (CR 67% and PR 27%)
Adverse events					
B‐cell aplasia	All patients with response to treatment	Only one patient had normal B‐cell count before infusion	25% had persistent B cell aplasia at 24 months post infusion	92% had B cell aplasia at baseline	None
CRS, *n* (%)	58 (77)	64 (58)	100 (92)	113 (42)	62 (91)
Neurologic events, *n* (%)	30 (40)	23 (21)	72 (67)	80 (30)	43 (63)
Pyrexia, *n* (%)	30 (40)	39 (35)	94 (87)	45 (17)	62 (91)
Febrile neutropenia, *n* (%)	27 (36)	18 (16)	39 (36)	25 (9)	Not reported
Hypotension, *n* (%)	22 (29)	29 (26)	63 (58)	60 (22)	35 (51)
Elevated AST, *n* (%)	20 (27)	Not reported	19 (18)	Not reported	21 (31)

In this review, we focus on recent advances in the development of CAR‐engineered NK cells as an alternative immune cell effector for the treatment of different malignancies, highlighting advantages over CAR T‐cell therapy and the preclinical and clinical milestones that have been achieved so far.

## Advantages of NK cell over T cells for CAR engineering

CD8^+^ T cells are effector lymphocytes whose function depends on the activation of the T‐cell receptor (TCR) after it recognises a specific antigen bound to a major histocompatibility complex (MHC) class I on the surface of target cells.^13^, ^14^ Thus, CD8^+^ T‐cell activation depends on a first specific signal generated by the TCR, leading to secretion of granzyme and perforin, which then kill malignant or infected cells.^13^ Although NK cells share certain cytotoxic functions with CD8^+^ T cells, they do not require prior antigen priming before they are able to kill their targets. Instead, NK cells rely on the balance between activating and inhibitory inputs generated by several germline‐encoded receptors for their cytotoxic functions towards pathogens and transformed cells.^15^, ^16^ Once equipped with CARs, they maintain their ability to be triggered by these innate receptors, while antigen recognition is redirected towards CAR‐specific targets. This feature, absent in T cells, preserves NK cell‐mediated cytotoxicity towards malignant cells, even in the event of target antigen loss or downregulation.

Thus far, the approved CAR T‐cell products have been autologous because of the risk of GVHD with the use of allogeneic T cells.^17^ However, harvesting autologous T cells from cancer patients can be difficult as a result of lymphopenia induced by multiple lines of therapy and/or high disease burden in patients with leukaemia. The autologous T cells must be transduced and expanded before infusion, a manufacturing process that can take precious days away from patients in need of urgent treatment.^18^ In contrast to T cells, allogeneic NK cells are not associated with GVHD^19^, ^20^, ^21^ and readily available as an allogeneic off‐the‐shelf product, making them an attractive option for cellular therapy.

Finally, CAR T‐cell therapy has been associated with serious clinical adverse events such as CRS and ICANS.^10^, ^22^ In fact, in the phase I and phase II clinical trials that led to the approval of CD19‐targeted CAR T‐cell therapy, CRS of any grade was experienced by 58–92% of patients, while neurologic events of any grade were seen in 21–67% of patients who received an infusion.^5^, ^6^, ^7^ Recently, our group has shown that CAR‐NK cells lack measurable toxicity in patients with non‐Hodgkin lymphoma and chronic lymphocytic leukaemia.^23^

## Potential sources of NK cells for CAR engineering

**Figure 1:**
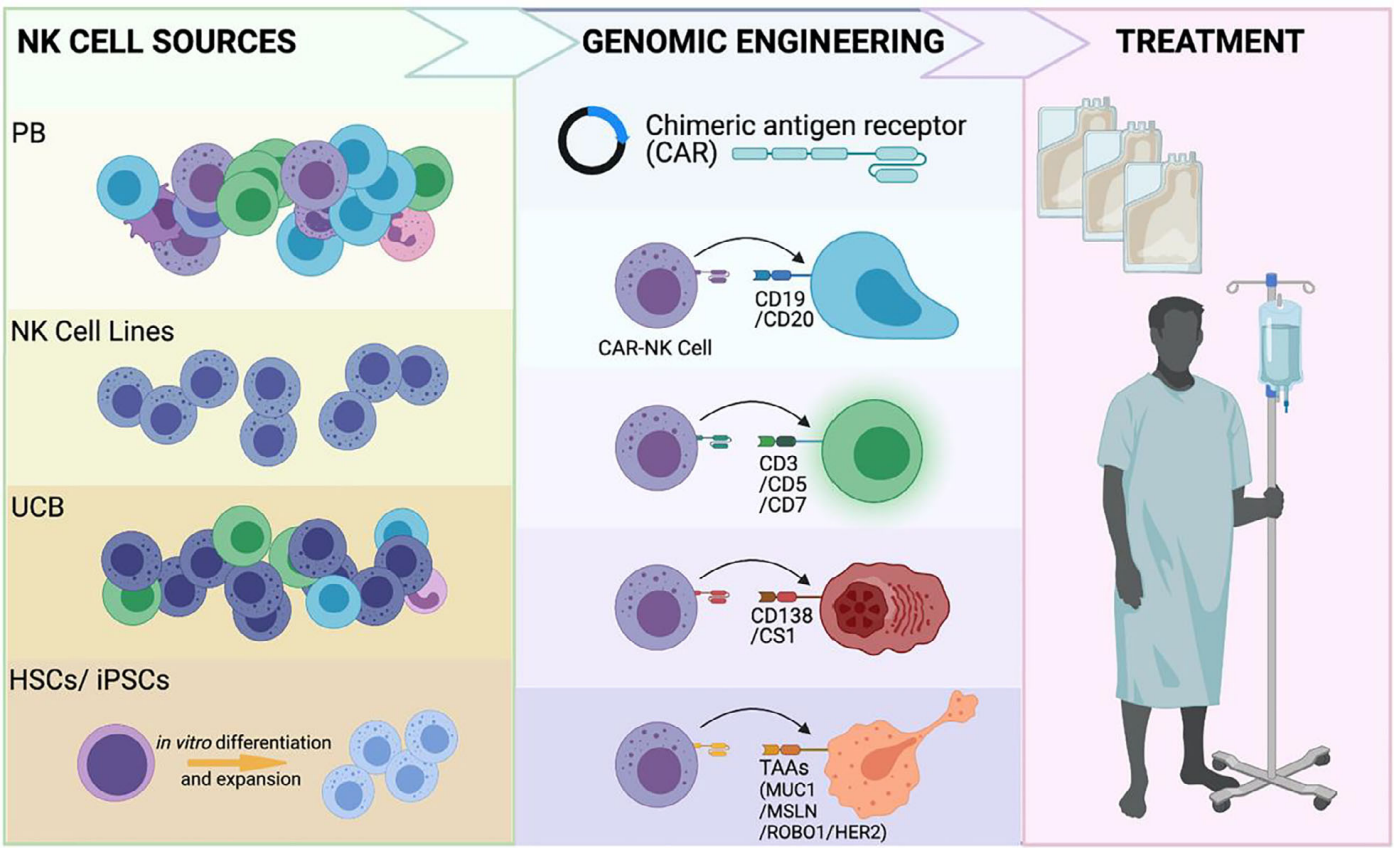
CAR‐NK cells for antitumor therapy. HSCs, haematopoietic stem cells; iPSCs, induced pluripotent stem cells; MSLN, mesothelin; PB, peripheral blood; TAA, tumor‐associated antigen; UCB, umbilical cord blood.

NK cells can be derived from multiple sources for use in CAR engineering, as discussed below and shown in Table 2 and Figure 1.

**Table 2:** Pre‐clinical studies on CAR NK therapy

Study	Target	Malignancy	Stimulatory and co‐stimulatory domains	Source of NK cells	Culture and expansion	Engineering method
Liu *et al*.^23^	CD19	B‐cell malignancies	CD3ζ and CD28	Cord blood	Genetically modified K562 feeder cells co‐expressing IL‐21 and CD137, ectopic IL‐15 and exogenous hrIL‐2^a^	Retrovirus
Herrera *et al*.^66^	CD19	B‐cell malignancies	CD3ζ and 4‐1BB	Peripheral and cord blood	Exogenous hrIL‐2 and IL‐15	Lentivirus
Muller *et al*.^65^	CD19	B‐cell malignancies	CD3ζ and CD28	Peripheral blood	Exogenous IL‐15	Lentivirus or alpharetrovirus
Quintarelli *et al*.^64^	CD19	B‐cell malignancies	CD3ζ and 4‐1BB	Peripheral blood	NK Cell Expansion kit containing antibodies against NKp46 and CD2 and exogenous hrIL‐2 or IL‐15	Retrovirus
Goodridge *et al*.^67^	CD19	B‐cell malignancies	CD3ζ and 2B4	iPSC	Autonomous IL‐15 receptor	Unknown
Romanski *et al*.^60^	CD19	B‐ALL	CD3ζ	NK‐92	Exogenous hrIL‐2	Retrovirus
Oelsner *et al*.^62^	Flt3	B‐ALL	CD3ζ and CD28	NK‐92	Exogenous hrIL‐2	Lentivirus
Muller *et al*.^59^	CD20	B‐cell malignancies	CD3ζ	NK‐92	Exogenous hrIL‐2	Retrovirus
Tassev *et al*. ^63^	EBNA	EBV+ B‐lymphoblastic cell lines	CD3ζ and 4‐1BB	NK‐92MI^b^	No cytokines, feeder cells or stimulation beads	Retrovirus
Boissel *et al*.^61^	CD19	CLL	CD3ζ	NK‐92	Exogenous hrIL‐2	Electroporation
Jiang *et al*.^71^	CD138	Multiple myeloma	CD3ζ	NK‐92MI^b^	No cytokines, feeder cells or stimulation beads	Lentivirus
Chu *et al*.^73^	CS1	Multiple myeloma	CD3ζ and CD28	NK‐92	Exogenous hrIL‐2	Lentivirus
Chen *et al*.^76^	CD3	Peripheral T‐cell lymphoma	CD3ζ, CD28 and 4‐1BB	NK‐92	Exogenous hrIL‐2	Lentivirus
Chen *et al*.^75^	CD5	Peripheral T‐cell lymphoma	CD3ζ, CD28 and 4‐1BB	NK‐92	Exogenous hrIL‐2	Lentivirus
You *et al*.^77^	CD7	T‐ALL	CD3ζ, CD28 and 4‐1BB	NK‐92MI^b^	No cytokines, feeder cells or stimulation beads	Electroporation
Han *et al*.^48^	EGFR and EGFRvIII	Glioblastoma	CD3ζ and CD28	NK‐92 and NKL^c^	Exogenous hrIL‐2	Lentivirus
Murakami *et al*.^82^	EGFRvIII	Glioblastoma	CD3ζ, CD28 and 4‐1BB	KHYG‐1	Exogenous hrIL‐2	Lentivirus
Genssler *et al*.^81^	EGFR EGFRvIII^d^	Glioblastoma	CD3ζ and CD28	NK‐92	Exogenous hrIL‐2	Lentivirus
Zhang *et al*.^85^	HER2	Glioblastoma	CD3ζ and CD28	NK‐92	Exogenous hrIL‐2	Lentivirus
Muller *et al*.^82^	EGFRvIII	Glioblastoma	DAP12	NK cell line YTS	No cytokines	Lentivirus
Sahm *et al*.^85^	EpCAM	Breast cancer	CD3ζ and CD28	NK‐92	Exogenous hrIL‐2 and ectopic IL‐15	Lentivirus
Liu *et al*.^88^	HER2	Breast cancer	CD3ζ and CD28	NK‐92	Exogenous hrIL‐2	Electroporation
Chen *et al*.^89^	EGFR and EGFRvIII	Breast cancer brain metastases	CD3ζ and CD28	NK‐92	Exogenous hrIL‐2	Lentivirus
Liu *et al*.^90^	EGFR	Triple negative breast cancer	CD3ζ, 4‐1BB and CD28	Peripheral blood	Exogenous hrIL‐2	Lentivirus
Hu *et al*.^91^	Tissue Factor	Triple negative breast cancer	CD3ζ, 4‐1BB and CD28	NK‐92MI^b^	No cytokines, feeder cells or stimulation beads	Lentivirus
Li *et al*.^54^	Mesothelin	Ovarian cancer	CD3ζ and multiple co‐stimulatory domains including DAP10, 2B4 and 4‐1BB	NK‐92 iPSC	Exogenous hrIL‐2 After NK cell differentiation, feeder cells expressing membrane bound IL‐21 and exogenous hrIL‐2	Transposon plasmids
Uherek *et al*.^92^	HER2	Ovarian and breast cancer	CD3ζ	NK‐92	Exogenous hrIL‐2	Retrovirus

B‐ALL, B‐cell acute lymphoblastic leukemia; CLL, chronic lymphocytic leukemia; iPSC, induced pluripotent stem cell; T‐ALL, T‐cell acute lymphoblastic leukemia.

^a^ hrIL‐2: human recombinant interleukin‐2.

^b^ NK‐92MI: genetically modified NK‐92MI cells produce high levels of IL‐2 and possess a high‐affinity CD16 variant.

^c^ NKL is cell line derived from an adult with NK cell leukemia.

^d^ Dual specific NK cells targeting wildtype EGFR and mutated EGFRvIII.

### Peripheral blood NK cells

NK cells (classically defined by flow cytometry as CD56‐positive, CD3‐negative, CD19‐negative, CD14‐negative and CD45‐positive^24^) are short‐lived innate lymphoid cells with a turnover time of about 2 weeks in the circulation at steady state.^25^, ^26^ They typically represent 10–15% of peripheral blood (PB) lymphocytes,^27^, ^28^ but their frequency can vary widely among healthy individuals, from 0% to 60% PB‐NK cells (CD3^−^CD56^+^ cells) in one study.^27^ NK cells can be divided into two major subsets according to their phenotype: CD56^bright^CD16^dim^ or CD56^dim^CD16^bright^ cells, the latter being the most frequent in PB.^27^ Unlike T cells, NK cells do not display rearranged receptors such as TCRs. Hence, their diversity reflects the combination of germline‐encoded activating (e.g. NKG2C, NKG2D and natural cytotoxic receptors such as NKp30, NKp44 and NKp46) and inhibitory (e.g. killer immunoglobulin‐like receptors [so‐called KIRs] and NKG2A) receptors on their surface.^29^ Human PB‐NK cells are typically characterised by a mature cytotoxic CD56^dim^CD16^bright^ subset^30^ with little variation among individuals.^25^ Overall, however, PB‐NK cell phenotypic diversity seems to be donor‐specific and highly variable among individuals.^25^ Furthermore, while inhibitory receptors are mainly genetically regulated to retain self‐tolerance, NK cell‐activating receptors adapt to diverse environmental stimuli in response to pathogens and malignancies.^29^ Finally, PB‐NK cell functionality and diversity can be modulated by exposure to cytokines such as interleukin (IL)‐2, IL‐15, IL‐18, IL‐12 and IL‐21.^25^, ^31^

In summary, as a candidate source of an off‐the‐shelf product, PB‐NK cells possess the advantages of easy accessibility and mature phenotypic signature. However, donor variability complicates dose standardisation as PB‐NK cells lack a single and uniform renewable source.^32^

### Umbilical cord blood NK cells

NK cells can also be derived from umbilical cord blood (CB). In fact, their absolute number in CB by volume and fraction is higher than found in PB, representing 20–30% of the CB lymphocytic pool.^28^ On the other hand, CB‐NK cells show lower diversity and display a more immature phenotype than do PB‐NK cells.^30^, ^33^ The CB‐NK cell phenotypic signature features a higher expression of CD56^bright^, NKG2A, CD94, c‐kit (CD117), Trail, CD62L and CD27, together with a lower expression of CD16, KIRs, NKG2D, NKG2C, TIGIT and DNAM‐1.^30^, ^33^ The transcriptome of CB‐NK cells also shows lower levels of maturation markers such as T‐bet, eomesodermin, perforin and granzymes.^30^, ^33^ Consistent with these data, when freshly isolated without any *ex vivo* expansion, CB‐NK cells function poorly in co‐cultures with tumor cells, as evidenced by their lower cytokine production and diminished degranulation together with decreased cytotoxicity against tumor targets.^30^, ^33^

Despite their initial immature profile, CB‐NK cells can be successfully expanded and induced to develop more mature characteristics by exposure to cytokines. For example, when cultured in lower concentrations of IL‐2 (200 IU), CB‐NK cells fail to proliferate, probably because of their lower CD25 expression.^33^ However, when exposed to higher concentrations of IL‐2 (1000 IU), their proliferation rates improve significantly together with their degranulation and cytotoxic capacity.^33^ CB‐NK cell expansion can be further improved by co‐culture with ‘feeder cells’ such as genetically modified K562 cells (erythroleukaemia cell line) that express membrane‐bound IL‐21, 4‐1BB ligand and CD48, which increases the proliferation rate by more than 1000‐fold.^34^ Finally, CB‐NK cells can be successfully transduced after expansion with ‘feeder cells’ and/or cytokines. After transduction with a CD19‐directed CAR, the expanded CB‐NK cells show enhanced metabolic fitness and greater cytotoxicity against CD19‐positive tumor than in non‐transduced NK cells.^34^, ^35^

Thus, CB units offer a rich source of NK cells for immunotherapy. They are readily available in global CB banks, can be expanded using feeder cells and cytokines, and genetically manipulated to express CARs. Nonetheless, similar to PB‐NK cells, their phenotype and yields differ significantly among donors, and they lack a single renewable source.^32^

### NK cells from umbilical cord CD34^+^ progenitors

NK cells can also be generated from CD34^+^ progenitor cells isolated from umbilical CB. This allows the production of a more homogenous and well‐defined NK cell product than does the use of PB‐NK or CB‐NK cells.^36^ CD34^+^ haematopoietic progenitors can be successfully differentiated into CD56^+^ NK cells and then expanded with cytokines in a stepwise fashion.^37^ It should be stressed, however, that cells derived by this method do not seem to be as mature as PB‐NK cells, as they express high levels of CD56, NKG2A and CD94, and display variable KIR levels.^38^ Moreover, despite their cytotoxic competency against leukaemic cells,^39^ these CD56^+^ cells show low expression of CD16 (FcγRIII), resulting in poor antibody‐dependent cellular cytotoxicity (ADCC).^40^ To the best of our knowledge, only one study has successfully introduced a CD19‐targeted CAR into CD34^+^ progenitors followed by NK differentiation.^41^

Hence, although CD34^+^ cells from CB can be used as a source for large‐scale production of a homogenous population of CD56^+^ NK cells, they are immature and display little ADCC capacity even after expansion, thus requiring additional steps to enhance potency for clinical application.

### NK‐92 cell line

To avoid some of the difficulties in handling primary NK cells, investigators have turned their efforts to developing a safe immortalised NK cell line for cellular therapy. NK‐92 cells, for example, were derived from a 50‐year‐old patient who had a large granular lymphocyte non‐Hodgkin lymphoma characterised by a CD56^+^, CD3^−^ and CD16^−^ population.^42^ These cells, which are IL‐2‐dependent, have cytolytic activity against K562 (erythroleukaemia) and Daudi (Burkitt lymphoma) cell lines.^42^ Infusing cells derived from a cancer patient might appear to be a perilous alternative; however, when irradiated, these cells have no malignant potential, neither in mice nor in humans, and they maintain their cytotoxic capacity.^43^ Administration of irradiated NK‐92 cells seems safe with no significant adverse effects,^44^ and these cells can be effectively engineered to improve their function. For example, NK‐92 cells do not express CD16 (FcγRIIIa), the main receptor involved in ADCC and a powerful activating signalling pathway that has been implicated in NK cell cytotoxic capacity. However, these cells can be genetically modified to express a high‐affinity CD16 receptor variant that improves their antitumor performance in the presence of diverse monoclonal antibodies.^45^ In addition, NK‐92 cells can be modified to express CARs, a strategy that is being tested in a phase I/phase II clinical trials for diverse types of malignancies (NCT02892695 and NCT02742727). Unfortunately, the lifespan of these cells is quite short because of their pre‐infusion irradiation, which hinders their *in vivo* proliferation, an essential factor in tumor control.^32^ Less frequently, other NK cell lines derived from individuals with NK cell leukaemia, such as NKL,^46^ KHYG‐1^47^ and YTS,^48^ have been genetically manipulated to express CARs.

Thus, NK‐92 cells are a renewable, homogenous and genetically modifiable population of NK cells that constitute a potential source for off‐the‐shelf cellular therapy, keeping in mind that the clinical application of this product could be limited by its diminished antitumor potency (because of irradiation) when compared to NK cells from other sources.

### NK cells from induced pluripotent stem cells

NK cells can be successfully generated from human‐induced pluripotent stem cells (iPSCs) through a stepwise approach: first by co‐culturing iPSCs with bone marrow‐derived stromal cells followed by exposure to a specific cytokine cocktail to drive NK differentiation.^49^, ^50^, ^51^ Moreover, iPSC‐derived NK cells develop activating and inhibitory receptors (including KIRs, natural cytotoxic receptors and CD16) that are typical of their mature counterparts, and they demonstrate *in vitro* and *in vivo* antitumor activity.^49^, ^50^, ^52^ After differentiation of iPSCs into NK cells, they can be expanded by co‐culture with feeder cells, such as genetically modified artificial antigen‐presenting cells (aAPCs).^51^

Not surprisingly, iPSC‐derived NK cells have been genetically modified to express CARs. The first experiences with CAR introduction into iPSC‐derived NK cells were in the HIV infection domain, in which a CD4‐targeting CAR possessing a CD3ζ intracellular domain controlled viral replication *in vitro* and in mouse xenograft models.^53^ Chimeric antigen receptors directed to tumor antigens have also been transduced into iPSC‐derived NK cells to direct their activity and improve antitumor cytotoxicity.^54^

In summary, iPSC‐derived NK cells remain a compelling homogenous source of cellular therapy. These cells can be genetically manipulated and produced in large scale, showing mature phenotypic profile together with antitumor cytotoxicity.^52^, ^54^, ^55^ Nonetheless, they lack important activation markers that might limit their antitumor cytotoxicity.^56^ Hence, their efficacy in *in vivo* models and in the clinical setting remains to be shown.

**Figure 2:**
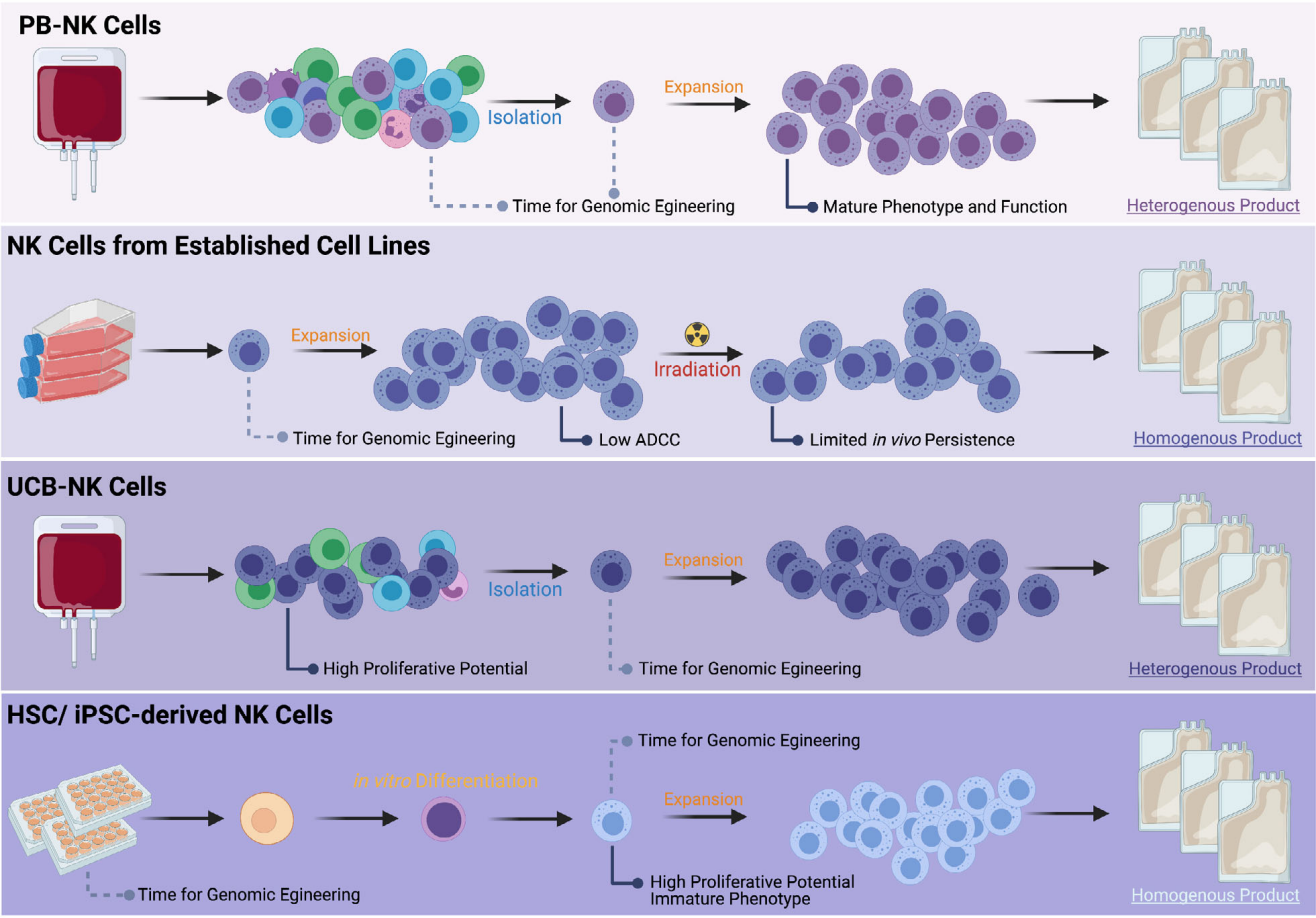
Different sources of allogeneic NK cells for engineering. ADCC, antibody‐dependent cellular cytotoxicity; HSC, haematopoietic stem cell; iPSC, induced pluripotent stem cell; PB, peripheral blood; UCB, umbilical cord blood.

To conclude this section, NK cells can be isolated, expanded and engineered from various sources using different methodologies, and each source has specific advantages and disadvantages as depicted in Figure 2.

## Preclinical studies of CAR‐engineered NK cells

The concept of improving NK cell function by modifying the cells to express a CAR molecule has great appeal, leading several research groups to test this strategy over the past decade, both *in vitro* and *in vivo* in mouse xenograft models of cancer. These studies have explored different sources of NK cells, alternative methods for NK cell culture, expansion and transduction, and different plasmid constructs and vectors (Table 2).

### B‐cell malignancies

NK cells from diverse sources have been genetically modified to express CARs to redirect their activity against B‐cell malignancies. So far, CD19 has been the most commonly surveyed antigen because of its enriched expression in malignant B cells and lack of expression in most normal cells.^57^, ^58^ Innovative approaches have been employed in the investigation of CAR‐NK cells for the treatment of B‐cell malignancies.

First‐generation CARs carrying a single CD3ζ costimulatory moiety have been introduced into NK‐92 cells using retroviral transduction.^59^, ^60^ Two studies have explored this approach with both adopting CD8 as the CAR hinge and transmembrane domain.^59^, ^60^ The difference between these CARs was their single‐chain variable fragment (scFV) specificity, as one targeted CD19^60^ and the other CD20.^59^ Boissel *et al*.^61^ also produced first‐generation CAR‐armed NK‐92 cells targeting CD19 to test against CLL cells. Their construct displayed a CD8 hinge and transmembrane domain, and the plasmid delivery was performed using electroporation.^61^ NK‐92 cells have also been engineered with second‐generation CARs. Oelsner *et al*.^62^ worked on a second‐generation construct carried by a lentiviral vector and possessing CD3ζ and CD28 stimulatory domains together with a CD28 transmembrane element followed by a modified CD8 hinge. To direct cytotoxicity against Fms‐like tyrosine kinase (Flt3)‐positive B‐ALL cells, they built a CAR immunoglobulin‐like domain specific for Flt3, which led to enhanced antitumor activity. These CAR‐engineered NK‐92 cells required consecutive administration of IL‐2 while in culture.

Another group redirected the antitumor activity of NK‐92MI cells, which are genetically modified NK‐92 cells that independently produce IL‐2 and express a high‐affinity CD16 variant.^63^ They did so by introducing into these cells a TCR‐like CAR targeting an HLA‐restricted Epstein–Barr virus (EBV)‐encoded nuclear antigen (EBNA). Their CAR scFv specifically recognised EBNA3C peptide bound to major histocompatibility complexes (MHCs) in EBV‐positive B‐ALL cells. In this study, the CAR hinge and transmembrane domain were CD8‐derived, and its costimulatory moieties were 4‐1BB together with CD3ζ. Retroviral vectors were used for the transduction of NK‐92MI cells. It should be noted that the strategies using NK‐92 subsets need pre‐infusion irradiation, which might decrease cell proliferation and persistence *in vivo*.

Moreover, three different studies have investigated the introduction of CD19‐targeted CARs into PB‐NK cells, all second‐generation CARs containing a CD3ζ moiety in addition to either 4‐1BB or CD28 intracellular domains.^64^, ^65^, ^66^ To link the extracellular immunoglobulin‐like element to the costimulatory domains, a CD8 hinge combined with either CD28 or CD8 transmembrane structures was used.^64^, ^65^ Lentivirus or retrovirus vectors were used to transduce these PB‐NK cells,^64^, ^65^, ^66^ which were successfully expanded by cytokine exposure including human recombinant IL‐2 and/or IL‐15.^65^, ^66^ Quintarelli *et al*.^64^ also added antibodies against NKp46 and CD2 to enhance NK cell stimulation. All these CD19‐targeted PB‐NK cells showed *in vitro* activity against B‐cell malignancies.^64^, ^65^, ^66^

iPSC‐derived NK cells have also been genetically modified to express CAR. Goodridge *et al*.^67^ introduced a CD19‐targeting CAR combined with an NKG2D transmembrane region together with a CD3ζ and a 2B4 stimulatory domains (NCT04245722). These iPSC‐derived NK cells also possessed a cytokine‐autonomous IL‐15 signalling through a recombinant fusion protein of IL‐15 and IL‐15 receptor alpha to enhance proliferation and persistence. This approach confers the advantage of cytokine independence to these modified cells. These CAR‐armed iPSC‐derived NK cells also display a high‐affinity non‐cleavable CD16 variant to improve ADCC and, in combination with rituximab, they showed enhanced *in vitro* antitumor cytotoxicity.

Our group has investigated fourth‐generation CB‐derived CAR‐transduced NK cells. After isolation and selection from CB, NK cells were co‐cultured with genetically modified K562‐based feeder cells expressing membrane‐bound IL‐21, 4‐1BB and SLAMF4 in IL‐2‐supplemented growth medium.^35^ This strategy promotes CB‐NK cell proliferation and expansion, providing a source for a large‐scale clinical product. Four to six days after isolation, the cells are transduced with a retroviral vector encoding a CD19‐targeting scFv, CD3ζ signalling domain along with CD28 costimulatory domain, IL‐15 to improve NK cell proliferation and persistence together with an inducible caspase‐9 (iCas9) to be used as a safety switch in the event of toxicity. These CB‐derived CAR‐NK cells showed improved proliferation and persistence together with enhanced *in vitro* and *in vivo* antitumor activity against B‐cell malignancies. We have also genetically manipulated NK cell checkpoints to enhance CAR‐NK cell fitness by deleting the cytokine‐inducible SH2‐containing protein (CIS) gene with CRISPR‐Cas9 technique. This strategy enhanced CAR‐NK cell aerobic glycolysis because of increased Akt/mTOR together with c‐MYC signalling resulting in better antitumor activity.^68^ These promising preclinical data were pivotal in the design of subsequent clinical trials testing the feasibility, safety and efficacy of CB‐derived CAR‐NK cells as cellular therapy for patients with B‐cell malignancies.

### Multiple myeloma

Despite the impressive advances in the treatment of multiple myeloma (MM), it still remains an incurable disease.^69^ As a result, the cellular therapy domain has been investigated and attempts have been made to develop CAR‐NK cells against MM.

CD138 (syndecan‐1) is a classical marker of plasma cells and highly expressed in MM. Its expression in MM cells is associated with enhanced proliferation, cell survival pathways and decreased apoptosis.^70^ NK‐92MI cells have been modified by lentiviral transduction to express a first‐generation CD138‐targeting CAR, carrying a CD3ζ intracellular domain and a flexible CD8 hinge portion. These CD138‐targeting CAR‐NK cells showed enhanced anti‐MM activity *in vitro* and in xenograft mouse models.^71^

Another candidate antigen for the treatment of MM is CS1 (SLAMF7 or CRACC), a surface glycoprotein highly expressed in plasma cells and MM.^72^ Chu *et al*. have worked on the introduction of a CS1‐specific CAR into NK‐92 cells. Using a lentiviral gene delivery system, they have created a second‐generation CS1‐targeting CAR‐NK‐92 comprising CD3ζ together with a CD28 intracellular stimulatory domain. These armed NK‐92 cells showed directed cytotoxicity against CS1‐positive MM cells and inhibited tumor growth in a xenograft MM model.^73^ However, one pitfall to this approach is that normal lymphocytes and NK cells also express CS1, which can result in fratricide not only *in vitro* but also *in vivo*, limiting NK cell numbers and their anti‐myeloma activity.

### T‐cell malignancies

T‐cell malignancies, including peripheral T‐cell lymphoma and T‐cell acute lymphoblastic leukaemia (T‐ALL), are haematologic malignancies with limited therapeutic options.^74^ Adoptive immune cell therapy with NK‐CAR engineering is a potential option in the therapeutic arsenal of these patients. Normal and malignant T cells express very specific antigens, such as CD3 and CD5, which are not expressed on NK cells and hence can be targeted by CAR‐NK cells.

So far, three third‐generation CAR NK cells have been investigated for the treatment of T‐cell malignancies. Chen *et al*. developed a CD5‐directed CAR NK‐92 cell possessing a CD8 hinge and transmembrane portions. Its intracellular element was composed of CD3ζ together with a 4‐1BB and a CD28 costimulatory domains.^75^ Using this same construct backbone, this group created a third‐generation CD3‐targeting CAR NK‐92 cells.^76^ These cells were transduced using a lentiviral system.^75^, ^76^ Lastly, You *et al*.^77^ have developed a CD7‐targeting CAR NK‐92 cells also bearing a CD3ζ intracellular domain together with a 4‐1BB and a CD28 costimulatory moieties. As opposed to the two previously described CAR NK‐92 cells, these CD7‐specific NK‐92 cells possess a Fc hinge and NK‐92 that were transfected by electroporation.^77^ The CD5‐, CD3‐ and CD7‐specific NK‐92 cells showed *in vitro* cytotoxicity against T‐ALL and T‐cell lymphoma cells and outperformed non‐transduced NK‐92 cells in a T‐cell leukaemia mouse xenograft model.^75^, ^76^, ^77^ CAR‐NK cells might be more suitable than CAR T cells for therapy of T‐cell malignancies because of their lack of expression of T‐cell antigens and consequently no fratricide.

### Solid tumors

Unfortunately, despite the remarkable success of CAR T cells in the treatment of haematologic malignancies, similar outcomes have not been observed in the therapy of solid tumors with modified T cells. These poor results are most likely because of the lack of adequate homing capacity together with the adverse immunosuppressive solid tumor microenvironment. In this context, several investigators have been researching the potential of CAR‐NK cell therapy against solid malignancies such as glioblastoma, melanoma, breast, ovarian and prostate cancers (Table 2).

Glioblastoma (GBM), a highly lethal primary brain tumor, expresses epidermal growth factor (EGFR), which is implicated in tumor proliferation and migration.^78^ This receptor can be detected at very low levels in normal brain tissue, and its gene amplification has been well characterised in the setting of GBM.^79^, ^80^ However, 30–40% of patients with GBM carry a mutant self‐active variant of EGFR, named EGFRvIII, which increases tumor aggressiveness.^80^ Both isoforms, EGFR and EGFRvIII, have been explored as antigen targets for CAR‐directed therapy. Han *et al*. developed a second‐generation CAR bearing a CD3ζ signalling and CD28 costimulatory domains together with an scFv region targeting both EGFR and EGFRvIII.^48^ These EGFR‐directed NK cells showed enhanced *in vitro* cytotoxicity, and their intracranial injection in an orthotopic mouse model resulted in improved tumor control and increased survival.^48^ Since both EGFR and its mutant form EGFRvIII have variable expression in GBM, Gressler *et al*. tested a novel dual‐targeting CAR construct targeted both EGFR and EGFRvIII and showed that NK‐92 cells expressing this CAR have superior antitumor cytotoxicity compared to their single targeting counterpart.^81^ Murakami *et al*. introduced a third‐generation EGFRvIII‐CAR into the KHYG‐1 NK cell line. Their EGFRvIII‐CAR KHYG‐1 NK cells carried a CD3ζ, CD28 and 4‐1BB intracellular domains and induced apoptosis in EGFRvIII‐positive GBM cells.^82^ Using a different NK cell line (YTS NK cell line), Müller *et al*. generated an EGFRvIII‐targeting NK cells with a DAP12 intracellular stimulatory domain. In addition, they also induced a constitutive expression of CXCR4 in these EGRFvIII‐CAR NK cells to improve their homing to the tumor. With this approach, they achieved tumor‐specific chemotaxis and directed tumor cytotoxicity.^83^ Lastly, the growth factor receptor tyrosine kinase Erb2 (HER2) is overexpressed in GBM tissue^84^ and has been explored as an alternative target for the treatment of patients with GBM. Zhang *et al*. introduced a second‐generation HER2‐directed CAR carrying CD3ζ in addition to a CD28 intracellular moiety. Their HER2‐CAR NK‐92 cells displayed enhanced cytotoxicity not only *in vitro* but also in xenograft mouse models.^85^ In all these preclinical GBM studies, lentivirus vectors were used for NK cell transduction. Hence, intracranially injected antigen‐directed CAR‐NK cells seem to be a promising option for the treatment of GBM.

Breast cancer is the most common malignancy among females in the United States. Not surprisingly, CAR‐NK cells have been investigated as an alternative therapeutic approach for this disease. Targeting diverse antigens, three breast cancer‐directed CARs carrying a CD3ζ intracellular domain together with a CD28 costimulatory moiety were introduced into NK‐92 cells. In this setting, the epithelial cell adhesion molecule (EpCAM) has been explored as a target antigen being overexpressed in carcinomas.^86^ Sahm *et al*. generated EpCAM‐targeting NK‐92 cells, and the svFc element of their CAR was linked to a CD8 hinge followed by a CD28 transmembrane domain. Moreover, their NK‐92 cells were armed with a membrane‐bound IL‐15 molecule capable of trans‐binding to the IL‐15 receptor on the cell surface to improve NK cell proliferation and persistence. These genetically modified NK cells showed enhanced tumor killing *in vitro*.^87^ On the other hand, Liu H *et al*. developed a second‐generation HER2‐directed CAR for the treatment of breast cancer. Their modified NK‐92 cells demonstrated specific *in vitro* cytotoxicity and, when injected into a xenograft breast cancer mouse model, these animals showed prolonged survival.^88^ Chen *et al*. created a second‐generation CAR with an scFv portion specific to both EGFR and EGFRvIII. Using a xenograft mouse model of breast cancer brain metastasis, they combined the intracranial injection of both their armed NK‐92 cells and an oncolytic virus to enhance tumor immunogenicity and consequently NK cell activation. Delayed tumor growth was observed with the combination of these two treatments.^89^ Different from the previous groups, Liu *et al*. created a third‐generation EGFR‐directed CAR carrying a CD3ζ portion together with CD28 and 4‐1BB costimulatory domains for the treatment of triple‐negative breast cancer. Their CAR had a CD8 hinge linked to a CD28 transmembrane element. These EGFR‐targeting CAR NK cells were directly injected into the tumor in both a cell line‐derived and a patient's tumor‐derived breast cancer mouse model, and they were able to better control tumor growth than in controls.^90^ Finally, Hu *et al*. developed an innovative CAR‐NK cell approach for the treatment of triple‐negative breast cancer, which consisted of an scFv portion targeting tissue factor (TF) on cancer cells. This TF‐specific CAR carried a CD28 transmembrane domain linked either to an IgG hinge or directly to the scFv element. Intracellular stimulatory domains comprised CD3ζ, CD28 and 4‐1BB moieties. These TF‐directed CAR‐NK‐92MI cells hampered tumor growth in a breast cancer xenograft mouse model.^91^

Additionally, some investigators have turned their attention to the investigation of CAR‐NK‐based therapies for the treatment of ovarian cancer. Uherek *et al*. developed first‐generation CAR NK‐92 cells directed to HER2, carrying a CD8 hinge and a CD3ζ intracellular stimulatory domain. These cells showed cytotoxicity specific to HER2‐positive cells and controlled tumor growth in mouse xenograft models.^92^ Li *et al* created human mesothelin‐targeting NK cells for the treatment of ovarian cancer. They initially screened different combinations of this scFv domain with diverse transmembrane and intracellular domains by introducing the CARs into NK‐92 cells. The transmembrane elements investigated were NKG2D, NKp44, NKp46, CD16 and CD28, while the stimulatory domains studied were CD3ζ, 4‐1BB, 2B4, DAP10 and DAP12. The best CAR‐NK cell performers had NKG2D transmembrane domain combined with CD3ζ and 2B4 stimulatory moieties, and two of them carried either an additional 4‐1BB or DAP10. Further investigations introduced the three most cytotoxic CARs into iPSC‐derived NK cells. iPSC‐derived NK cells carrying the combination of NKG2D transmembrane domain in addition to CD3ζ and 2B4 showed better performance *in vitro* and, when tested in animal models, showed enhanced antitumor activity.^54^

In summary, innovative CAR‐NK cell strategies have been investigated for the treatment of solid malignancies. Hopefully, preclinical data will be translated to the clinic to improve the therapeutic arsenal of patients with non‐haematologic cancers.

## CAR‐NK cells in the clinic

Given the impressive outcomes of CD19‐targeted T‐cell therapy in patients with B‐cell malignancies,^6^, ^7^, ^8^ seven phase I/phase II trials are currently investigating CAR NK cell treatment in this patient population. Three of these studies, not yet recruiting, were designed to target CD19 or CD22 (or both) in patients with relapsed or refractory B‐cell lymphoma (trials NCT03692767, NCT03690310, NCT03824964 and NCT04639739). The source of NK cells, signalling domains and vectors used for these studies are not disclosed. Two other trials, one completed (NCT00995137) and the second suspended for interim review (NCT01974479), are investigating the infusion of haploidentical second‐generation CAR NK cells targeting CD19 and possessing CD3ζ and 4‐1BB signalling domains in patients with relapsed or refractory B‐ALL. In the latter, electroporation was used to introduce the plasmid into expanded NK cells. Finally, one study using an iPSC‐derived CAR NK cell product targeting CD19, bearing 2B4‐CD3ζ intracellular domains and expressing a high‐affinity non‐cleavable CD16 to enhance ADCC potential along with a recombinant fusion of IL‐15 and IL‐15 receptor alpha to improve persistence,^67^ is now recruiting patients (NCT04245722).

There are also clinical trials testing CAR NK cells for patients with solid tumors. In this setting, a dose‐escalation phase I study is recruiting patients with metastatic solid tumors to investigate the safety of autologous or allogeneic NKG2D ligand‐targeted CAR‐NK cells transfected via mRNA electroporation (NCT03415100). Additional trials are exploring the infusion of CAR NK cells targeting distinct tumor antigens such as Muc1 for multiple solid cancers (NCT02839954), PSMA for castrate‐resistant prostate cancer (NCT03692663), mesothelin for epithelial ovarian cancer (NCT03692637) and ROBO1 for various solid tumors (NCT03940820 and NCT03941457). The vectors used to transduce NK cells in these studies were not disclosed.

The first‐in‐human clinical trial of CB‐derived CAR‐NK cells to treat patients with relapsed or refractory B‐cell haematologic malignancies (NCT03056339) was led by our group.^23^ In this dose‐escalation phase I/phase II clinical study, we are evaluating the safety and efficacy of CB‐NK cells modified to express anti‐CD19CAR, IL‐15 and inducible caspase‐9 (anti‐CD19CAR.IL15.iCas9). Eleven heavily pretreated patients with CD19‐positive cancers, including non‐Hodgkin lymphomas (NHL) and chronic lymphocytic leukaemia (CLL), have been treated with a single infusion of CAR NK cells following lymphodepletion chemotherapy. The overall response rate was 73% (8 of 11 patients) including 64% (7 of 11 patients) with complete responses and one case with resolution of Richter's transformation but persistence of CLL. Response durations could not be assessed owing to the study design as patients were allowed to receive consolidation such as a transplant once they had achieved a response. In contrast to large trials of CD19‐directed CAR T‐cell therapy (Table 1), none of our patients showed evidence of neurotoxicity, CRS, haemophagocytic lymphohistiocytosis or GVHD, nor were any admitted to the intensive care unit because of toxicities related to CAR‐NK cell treatment. A quantitative real‐time polymerase chain reaction assay, used to measure the expansion and persistence of CAR‐NK cells, detected these cells for at least 12 months in some patients.^23^ These encouraging results ratify the clinical feasibility, short‐term efficacy and safety of CAR‐engineered CB‐NK cell therapy.

Some clinical trials are currently testing CAR‐modified NK‐92 cells for the treatment of both haematologic and solid cancers. In the haematologic cancers, CAR NK‐92 cells targeting BCMA, CD7, CD19 and CD33 are being investigated as a therapeutic option for patients with multiple myeloma (NCT03940833), CD7‐positive haematologic malignancies (NCT02742727), CD19‐positive lymphoid malignancies (NCT02892695) and CD33‐positive acute myeloid leukaemias (NCT02944162). All are single‐armed unblinded phase 1 and phase 2 trials for patients with relapsed or refractory diseases. Both the CD7‐ and the CD33‐targeting CAR‐NK cells have CD28, 4‐1BB and CD3ζ as their signalling domains. One phase 2 open‐labelled clinical trial in the solid tumor domain is recruiting patients with HER2‐positive neuroblastoma to receive intracranial injections of HER2‐directed CAR NK‐92 cells carrying CD3ζ and CD28 signalling components (NCT03383978).

In summary, the preclinical and early clinical data described above are promising and support the application of NK cells as a useful platform for CAR engineering to target a range of malignancies.

## Conclusions and future directions

Genetically engineered immune effector cells equipped with CARs are potent additions to the immunotherapeutic arsenal against cancer. CAR T cells have produced remarkable clinical results in patients with relapsed or refractory B‐cell malignancies and multiple myeloma and are beginning to make inroads against solid tumors. Nonetheless, this therapeutic strategy has a number of limitations that underscore the need to identify alternative sources of immune cells, not only to overcome the safety issues, but also to lower the cost of such therapies and increase their accessibility to patients. Among the top candidates in the search, NK cells are especially promising. They have proved safe, technically feasible and potent in patients with lymphoid malignancies although a number of questions remain to be elucidated in future studies, including (1) the durability of the response, (2) the susceptibility of these cells to different suppressive elements in the tumor microenvironment, (3) their ability to overcome immune checkpoints and (4) their long‐term memory following immune encounters with tumor. Nevertheless, advances in engineering and gene‐editing techniques and new insights from the field of immunometabolism promise to address some of these issues and put CAR‐NK cells on the map as a clinical option for the treatment of haematologic and solid malignancies.

## Conflict of interest

KR, MD and The University of Texas MD Anderson Cancer Center (MDACC) have an institutional financial conflict of interest with Takeda Pharmaceutical for the licensing of the technology related to CAR‐NK cell research reported here. MD Anderson has implemented an Institutional Conflict of Interest Management and Monitoring Plan to manage and monitor the conflict of interest with respect to MDACC's conduct of any other ongoing or future research related to this relationship. KR and The University of Texas MD Anderson Cancer Center have an institutional financial conflict of interest with Affimed GmbH. Because MD Anderson is committed to the protection of human subjects and the effective management of its financial conflicts of interest in relation to its research activities, MD Anderson is implementing an Institutional Conflict of Interest Management and Monitoring Plan to manage and monitor the conflict of interest with respect to MD Anderson's conduct of any other ongoing or future research related to this relationship. KR participates on Scientific Advisory Board for GEMoaB, AvengeBio, Kiadis, GSK and Bayer.

## Author contributions

**May Daher:** Conceptualization; Writing‐original draft; Writing‐review & editing. **Luciana Melo Garcia:** Conceptualization; Writing‐original draft; Writing‐review & editing. **Ye Li:** Conceptualization; Writing‐original draft; Writing‐review & editing. **Katayoun Rezvani:** Conceptualization; Funding acquisition; Writing‐original draft; Writing‐review & editing.
